# Rheological properties of porcine organs: measurements and fractional viscoelastic model

**DOI:** 10.3389/fbioe.2024.1386955

**Published:** 2024-08-01

**Authors:** Aadarsh Mishra, Robin O. Cleveland

**Affiliations:** Institute of Biomedical Engineering, Department of Engineering Science, University of Oxford, Oxford, United Kingdom

**Keywords:** fractional viscoelasticity, elastography, porcine organs, rheology, biomechanics

## Abstract

The rheological properties of porcine heart, kidney, liver and brain were measured using dynamic oscillatory shear tests over a range of frequencies and shear strains. Frequency sweep tests were performed from 0.1 Hz to a maximum of 9.5 Hz at a shear strain of 0.1%, and strain sweep tests were carried out from 0.01% to 10% at 1 Hz. The effect of pre-compression of samples up to 10% axial strain was considered. The experimental measurements were fit to a Semi-Fractional Kelvin Voight (S-FKV) model. The model was then used to predict the stress relaxation in response to a step strain of 0.1%. The prediction was compared to experimental relaxation data for the porcine organ samples, and the results agreed to within 30%. In conclusion, this study measured the rheological properties of porcine organs and used a fractional viscoelastic model to describe the response in frequency and time domain.

## 1 Introduction

Biological materials exhibit time and history-dependent stress-strain viscoelastic behaviour (Fung, 1967; Nasseri et al., 2002). In the frequency domain, soft tissues exhibit a power law behaviour which suggests a broad range of relaxation processes that are likely present (Bonfanti et al., 2020). Fractional calculus can be used to model the power-law behaviour of linear viscoelastic models (Torvik and Bagley, 1984; Bagley and Torvik, 1986; Suki et al., 1994; Pritz, 1996; Fabry et al., 2001; Rossikhin and Shitikova, 2001) using elements known as springpots or Scott-Blair elements (Craiem and Armentano, 2006). Fractional viscoeleastic models have been applied to materials such as rubber, polymers and gels (Alcoutlabi and Martinez-Vega, 1998; Chen et al., 2004; Kobayashi et al., 2017; Aime et al., 2018; Bouzid et al., 2018; Kaplan et al., 2019; Katsourinis and Kontou, 2019; Bonfanti et al., 2020) and tissue such as the brain (Sack et al., 2009), liver (Kobayashi et al., 2005; Kobayashi et al., 2009; Nicolle et al., 2010; Kobayashi et al., 2012a; Kobayashi et al., 2012b), kidney (Nicolle et al., 2010), red blood cells (Craiem and Magin, 2010), and arteries (Craiem and Armentano, 2007; Craiem et al., 2008).

In this paper, viscoelastic properties of various porcine organs were measured and fit to a number of three-parameter biomechanical models. It was found that a semi-fractional Kelvin Voight (S-FKV) biomechanical model was the best fit to the power law behaviour of the organs up to 10% axial strain. Fractional viscoelastic models have previously been used in various studies (Kobayashi et al., 2005; Craiem and Armentano, 2007; Craiem et al., 2008; Kobayashi et al., 2009; Sack et al., 2009; Craiem and Magin, 2010; Nicolle et al., 2010; Kobayashi et al., 2012a; Kobayashi et al., 2012b), and the results reported here adds to the quantity of data available to researchers. Further in this study, the S-FKV model was fit to frequency domain measurements, and the model was then used to predict the time domain relaxation behaviour of the organs which were compared with the time domain experimental data. The parameters obtained from the S-FKV model were also extrapolated to higher frequencies and were comparable to elastography measurements.

The time scales (0.1–100 s) and strains (0.01%–10%) reported in these measurements are relevant to a number of clinical applications, in particular to provide input data for simulations. For example, elastography techniques which have been employed for diagnostic applications such as diagnosing liver fibrosis (Yin et al., 2007), characterising brain lesions (Kruse et al., 2008), and characterising cardiac diseases such as myocardial infarction (Kolipaka et al., 2009). A second example is minimally invasive surgery where needles (and other instruments) are inserted into tissue, such as: needle insertion into liver for radiofrequency ablation or percutaneous ethanol injection therapy (Kobayashi et al., 2009). In neurosurgery (Yundt et al., 1997), a biomechanical model could be used to optimise retractor-applied pressure and retractor position for reducing injury to tissue from retractor strains (Kyriacou et al., 2002). A final example is kidney stone treatment, both percutaneous nephrolithotomy done as key hole-surgery (Kallidonis et al., 2020; Barua et al., 2022) and shock wave lithotripsy (Pace et al., 2005) where the pulse repetition frequency of 1–2 Hz are typically employed.

## 2 Materials and methods

### 2.1 Preparation of porcine organ samples

**FIGURE 1:**
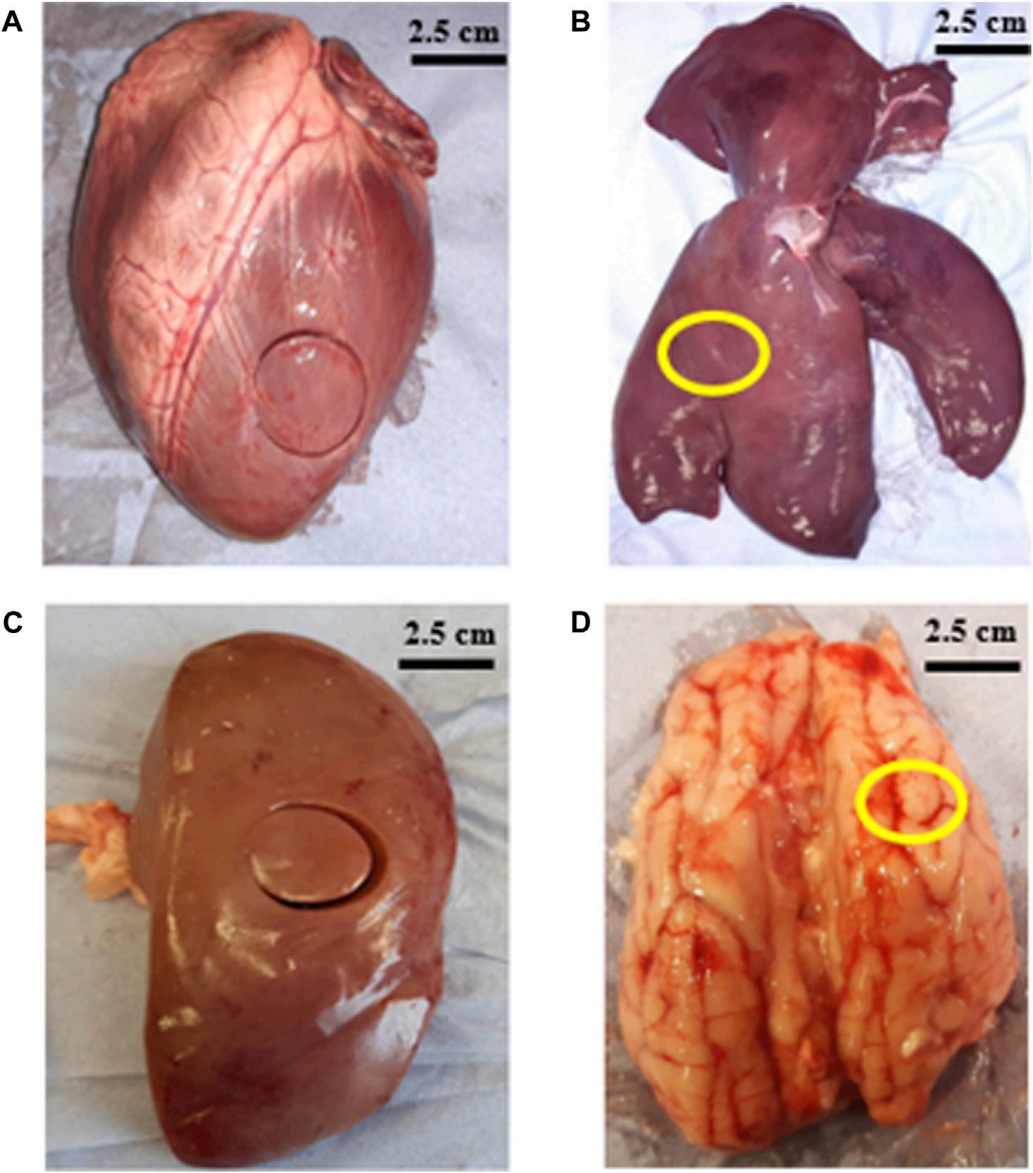
Images show porcine organs and location from where samples were removed **(A)** left ventricle of heart **(B)** right lobe of liver (marked in yellow) **(C)** middle region of kidney **(D)** frontal lobe of brain (marked in yellow).

Porcine heart, liver and brain were supplied by a local butcher 48–72 h after slaughter. Porcine kidneys were collected from a second local butcher 36–48 h after slaughter. All organs were tested within 2 h after collection. Figure 1 shows representatives images of the organs that were measured in the study. A cork borer was used to extract 25 mm diameter samples and surgical blades were used to dissect slices with 5–6 mm thickness (shown in Table 1). One sample per organ was extracted for all the tests.

**TABLE 1:** Thickness of porcine organ samples used for strain sweep, frequency sweep and relaxation tests.

Porcine organ	Sample thickness for strain sweep (mm)	Sample thickness for frequency sweep (mm)	Sample thickness for relaxation test (mm)
Heart (n = 4) [left and right ventricle]	5.9 ± 0.3 (n = 4)	5.9 ± 0.4 (n = 4)	6.0 ± 0.4 (n = 4)
Kidney (n = 13) [middle region]	5.0 ± 0.1 (n = 5)	5.3 ± 0.2 (n = 5)	5.4 ± 0.8 (n = 3)
Liver (n = 4) [left and right lobe]	5.8 ± 0.3 (n = 4)	5.3 ± 0.1 (n = 4)	5.8 ± 0.5 (n = 4)
Brain (n = 12) [frontal and parietal lobe of cerebrum along horizontal plane]	5.6 ± 0.6 (n = 4)	5.5 ± 1 (n = 4)	6.1 ± 1 (n = 4)

A stress controlled rheometer (Physica MCR 301) is a two plate system, where the upper plate applies torque to the sample and the lower plate is held fixed. The rheometer measures the torque applied and the angle of deformation, converts them into the shear stress and strain from which it outputs the storage modulus $G^{\prime }$ and loss modulus $G^{\prime \prime }$ . Sandpaper (200-grit size) was attached to the upper and lower plates of rheometer in order to minimise the slippage at the sample-plate interface. A custom made metallic casing was fixed to the bottom plate of rheometer and filled with Phosphate Buffered Saline (PBS) solution and maintained at 20°C.

### 2.2 Viscoelastic model

The springpot element employed in fractional calculus models can be thought of as part way in between a purely elastic element (spring) and a perfectly viscous element (dashpot) (Craiem and Magin, 2010). A fractional order derivative is used to represent its stress (σ) and strain (ε) relationship of a springpot:

$$\sigma =K_{\alpha }\;\frac{d^{\alpha }\varepsilon }{dt^{\alpha }}$$ (1)

where, *K*_α_ is the coefficient of consistence (with units of Pa.(s)^α^) and α is the order of fractional derivative (0 ≤ α ≤ 1). The bounding values of α represent the discrete elements employed in conventional viscoelastic models, that is a spring when α = 0 and *K*_α_ = *G* (modulus), and a dashpot when α = 1 and *K*_α_ = η (viscosity).

**FIGURE 2:**
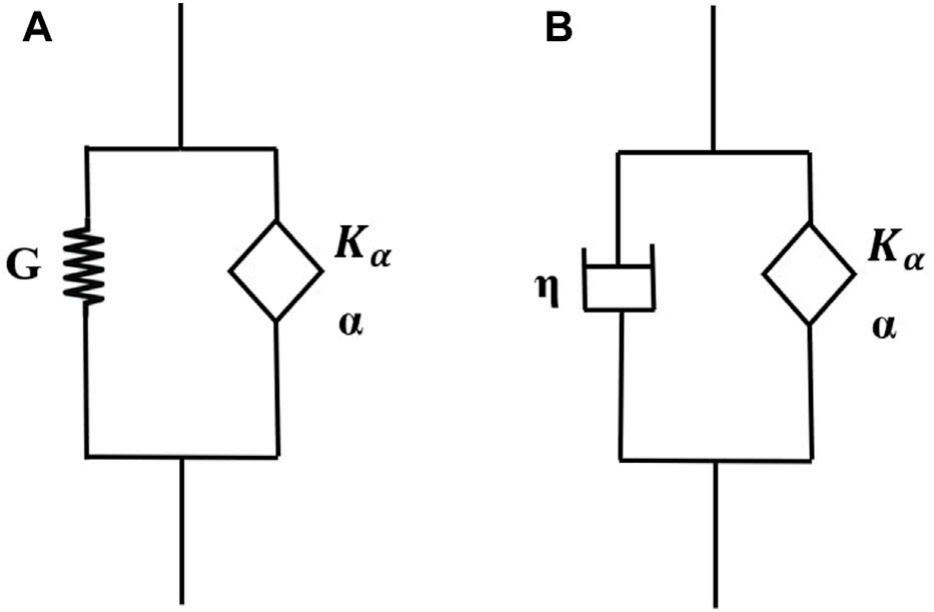
**(A)** Fractional Kelvin-Voight (FKV) model, and **(B)** Semi-Fractional Kelvin-Voight (FKV) model.

A fractional Kelvin Voight (FKV) model consists of a spring in parallel with a springpot (Figure 2A) and has a stress-strain relationship:

$$\sigma =K_{\alpha }\;\frac{d^{\alpha }\varepsilon }{dt^{\alpha }}+G\;\varepsilon$$ (2)

A S-FKV model consists of a springpot in parallel with a dashpot (Figure 2B) with the stress-strain relationship given by:

$$\sigma =K_{\alpha }\;\frac{d^{\alpha }\varepsilon }{dt^{\alpha }}+\eta \frac{d\varepsilon }{\mathrm{dt}}$$ (3)

In the frequency domain the modulus of the S-FKV can be expressed as storage modulus

$$G^{\prime }=K_{\alpha }\;\omega^{\alpha }\cos\frac{\alpha \pi }{2}$$ (4)

and a loss modulus

$$G^{\prime \prime }=K_{\alpha }\;\omega^{\alpha }\sin\frac{\alpha \pi }{2}+\eta \omega$$ (5)

## 3 Results

### 3.1 Strain sweep

**FIGURE 3:**
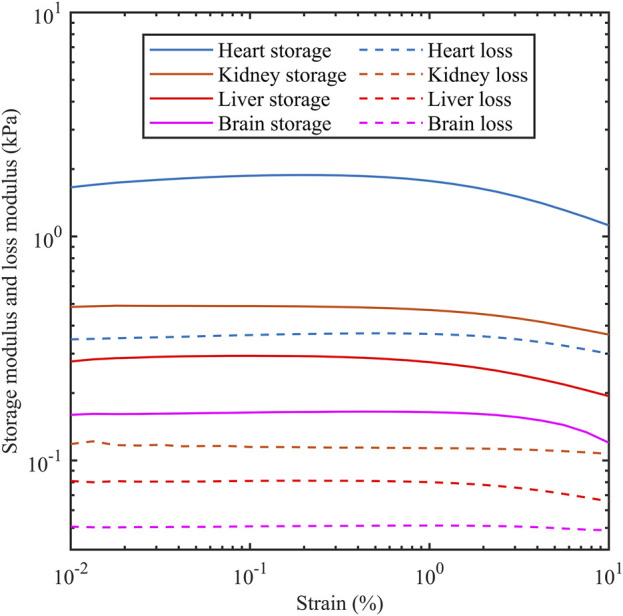
Storage modulus and loss modulus as a function of strain amplitude for porcine heart, kidney, liver and brain. Measurements were carried out at 1 Hz.

The storage modulus and loss modulus were provided by the rheometer using strain sweep tests as the strain amplitude was increased from 0.01% to 10% at a frequency of 1 Hz. Figure 3 shows the dependency of both moduli as a function of strain for different porcine organs. The horizontal sections are consistent with the tissue acting as a linear viscoelastic material which is up to 0.1% for the four organs tested here. Table 2 shows the storage modulus and loss modulus at 0.1% strain amplitude which is within the linear viscoelastic region.

**TABLE 2:** The storage modulus and loss modulus for different porcine organs at 0.1% shear strain, and 1 Hz frequency.

Porcine organs	Storage modulus (kPa)	Loss modulus (kPa)
Heart	1.87 ± 0.48	0.36 ± 0.01
Kidney	0.49 ± 0.03	0.11 ± 0.02
Liver	0.29 ± 0.08	0.08 ± 0.02
Brain	0.16 ± 0.03	0.05 ± 0.01

### 3.2 Frequency sweep

**FIGURE 4:**
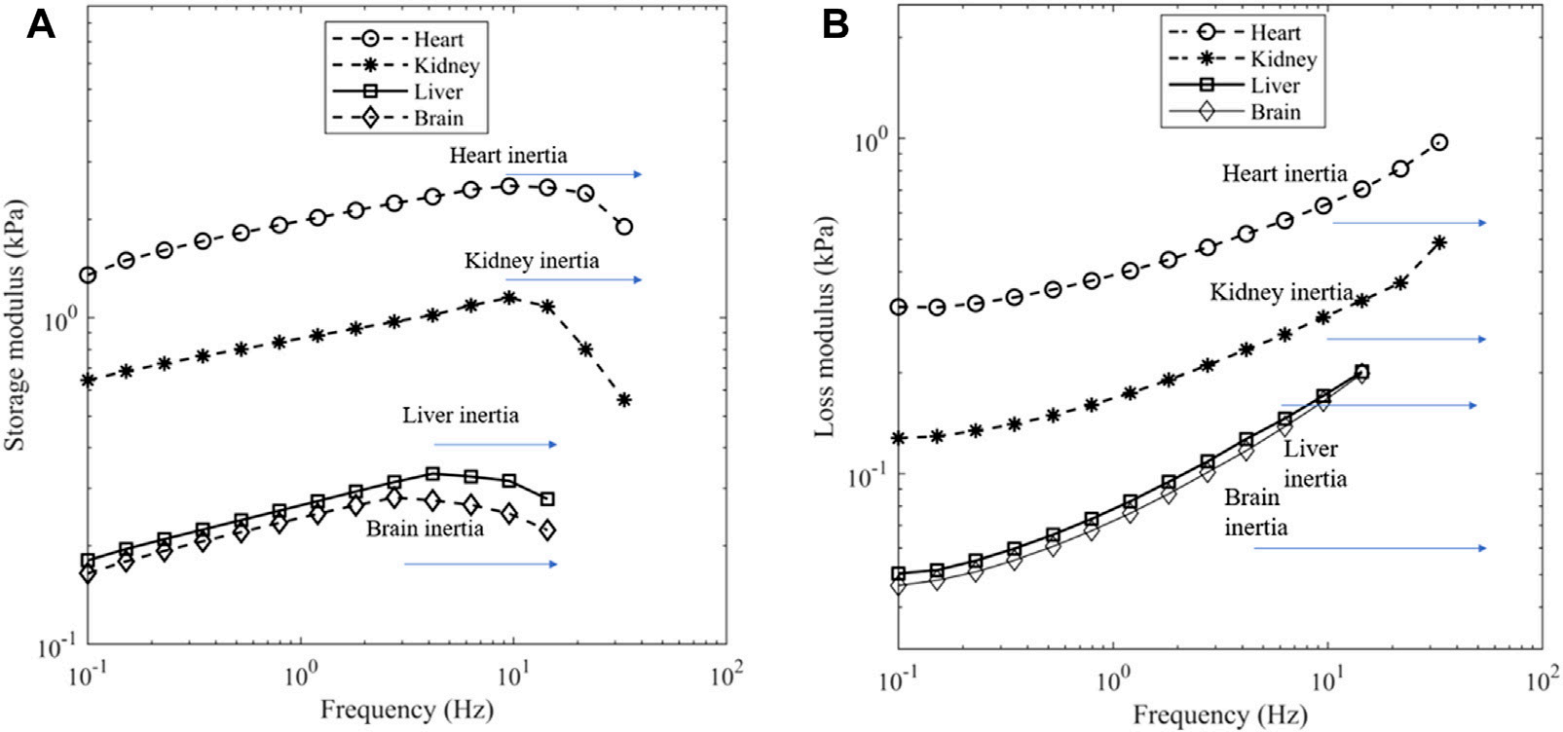
**(A)** Storage modulus and **(B)** loss modulus of porcine organs at a contact force of 0.1 N. Depending on the tissue, a dip in storage modulus can be seen near 9.5 Hz for heart and kidney, 4.2 Hz for liver and 2.8 Hz for brain which is indicative of inertia.

For the frequency sweep experiments, the strain amplitude was held at 0.1% and the frequency varied from 0.1 Hz until inertial effects appeared to impact the results. The presence of inertial effects can be seen by a reduction in storage modulus and an increase in the loss modulus (Ewoldt et al., 2015). Figures 4A, B show the effect, and the upper limit of frequency was 9.5 Hz for heart and kidney, 4.2 Hz for liver and 2.8 Hz for brain.

**FIGURE 5:**
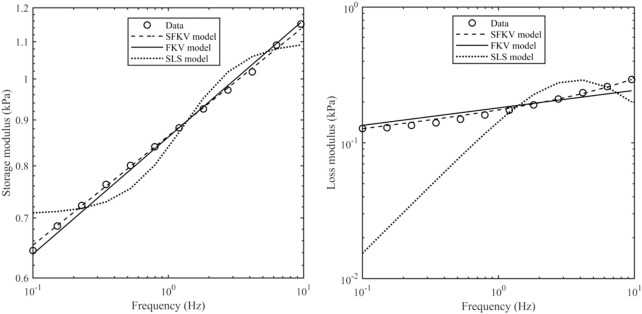
Storage modulus and loss modulus of porcine kidney at a contact force of 0.1 N along with the S-FKV, FKV and SLS model fits.

Figure 5 shows the experimental data from 0.1 to 9.5 Hz fitted to one of three models: a standard linear solid (SLS) model (spring in parallel with a spring and dashpot), a FKV model (springpot in parallel with a spring) and a S-FKV (springpot in parallel with a dashpot). Each model had three fitting parameters which were chosen using the least square fit function in MATLAB. It can be seen that that SFKV model best captures the response of the tissue and so the SFKV model used for the subsequent fitting on the manuscript.

For the rheometer measurements the samples were put in axial compression with a 0.1 N load before the shear moduli were recorded. In order to relate the axial load and axial strain the elastic modulus (E) was measured independently with a uniaxial compressive tests conducted on 15 porcine kidneys using a Universal Testing Machine (Instron 5582). For the kidneys the elastic modulus E∼44 kPa given by the slope of average stress-strain curve up to 2% axial strain (shown in Supplementary Material) is similar to 48.6 kPa reported in Ref. (Öpik et al., 2012). At a contact force of 0.1 N, the axial stress is σ≈ 200 Pa which for a typical elastic modulus results in an axial strain of 0.5%. For the other organs, the contact strain was determined using literature values for elastic modulus:110 kPa for porcine myocardium (Arunachalam et al., 2018), 17.5 kPa for porcine liver (Johnson et al., 2021) and 1.5 kPa for porcine brain [average of 1.2 kPa for gray matter and 1.8 kPa for white matter (Kaster et al., 2011)]. The contact force in those organs corresponded to an axial strain of 0.2% for the heart, 1.1% for the liver and 13.3% for the brain.

**FIGURE 6:**
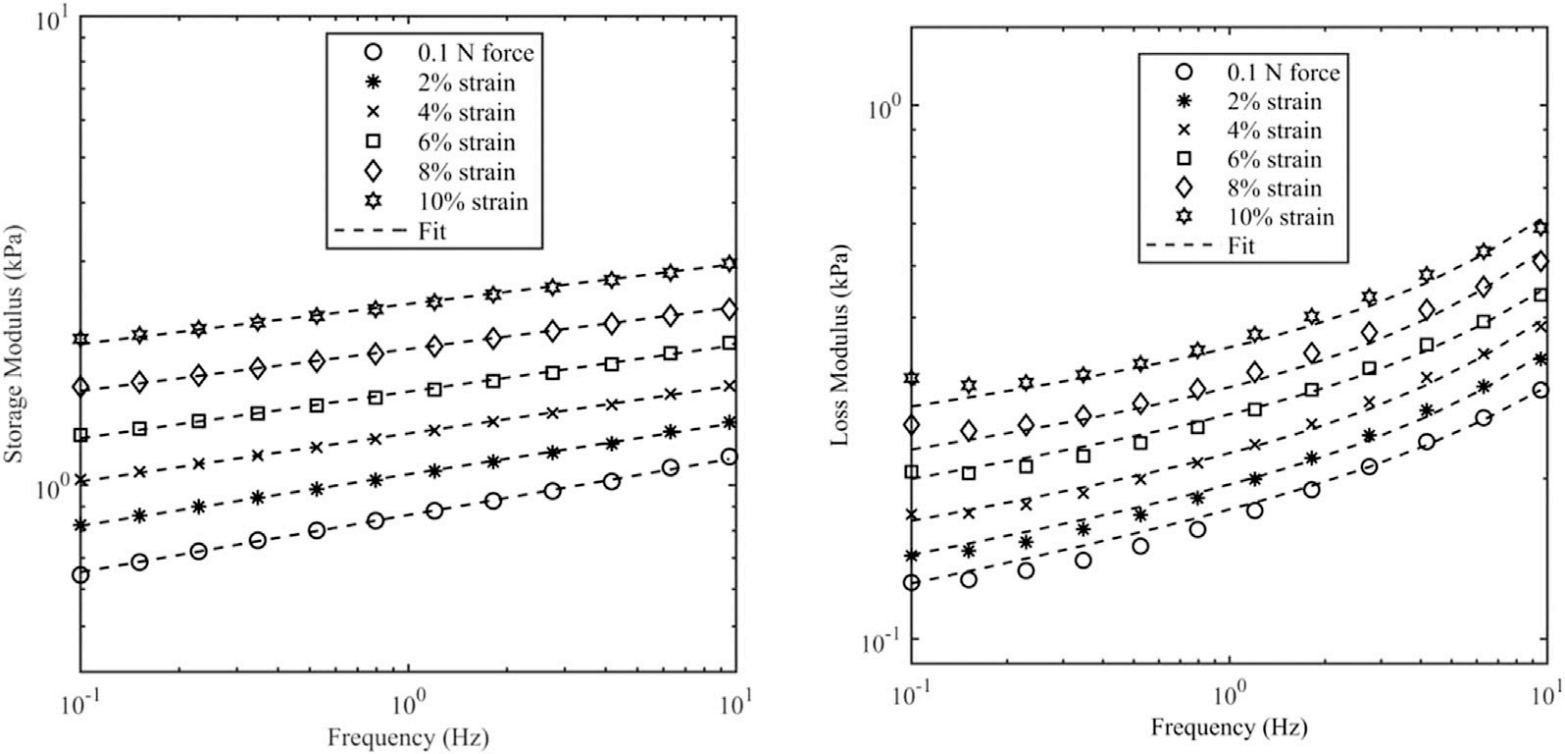
Storage modulus and loss modulus of kidney samples as a function of frequency and axial strain. Axial strain was varied up to 10%. The initial value was based on a contact force of 0.1 N which corresponds to 0.5% strain.

Figure 6 shows the data and model fit of the storage modulus and loss modulus of kidney samples for a range of axial strains, and it can be seen that there is a good agreement over the range of frequencies and strains considered here with a correlation coefficient better than 0.99. The raw data and fitted parameters for all organs are given in the Supplementary Material. Table 3 gives the fitted parameters of Eq. 3 in all porcine organs.

**TABLE 3:** S-FKV model parameters for different organs at a contact force of 0.1 N.

	$K_{\alpha }$ (kPa.(s)^α^)	α	η (Pa.s)
Heart	2.00 ± 0.64	0.13 ± 0.01	10.3 ± 6.00
Kidney	0.88 ± 0.25	0.12 ± 0.01	7.58 ± 3.00
Liver	0.27 ± 0.06	0.16 ± 0.02	9.70 ± 1.80
Brain	0.25 ± 0.03	0.16 ± 0.01	10.5 ± 2.50

**FIGURE 7:**
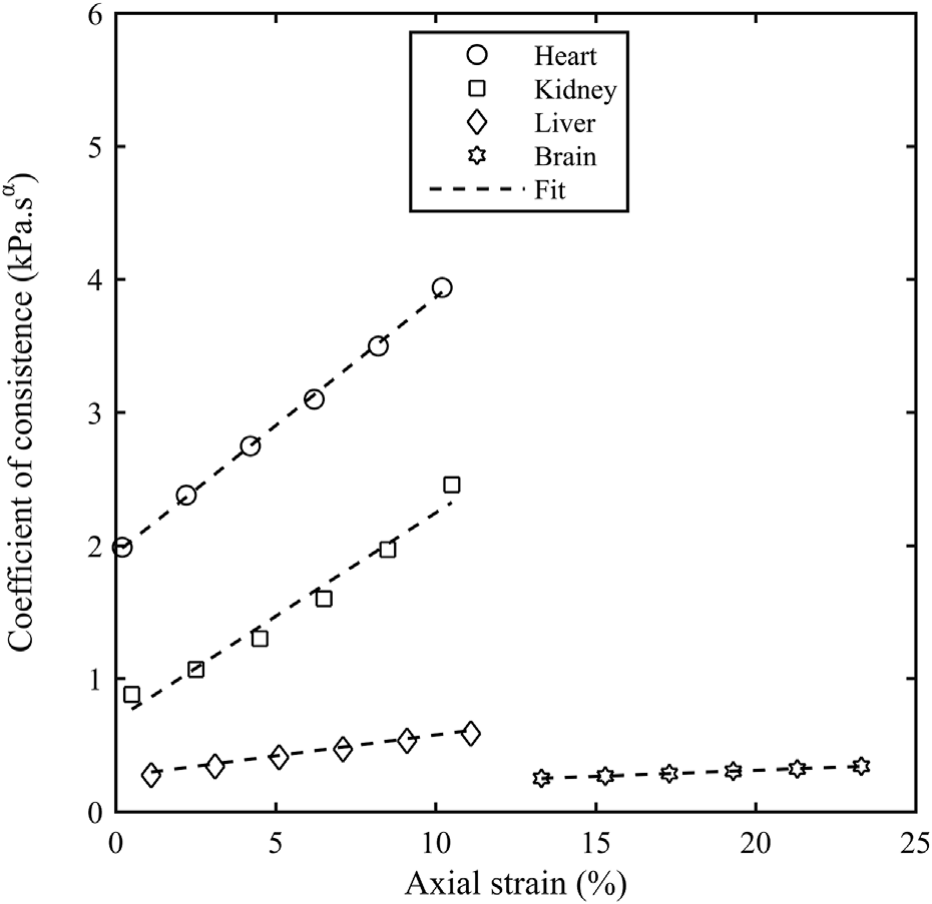
Coefficient of consistence (K_α_) as a function of axial strain (ε_A_) for different porcine organs. The contact force of 0.1 N corresponds to an axial strain of 0.2% for heart, 0.5% for kidney, 1.1% for liver and 13.3% for brain.

The storage modulus and the loss modulus increased monotonically with the axial strain $\left(\varepsilon_{A}\right)$ and frequency for all porcine organs. Figure 7 shows the dependence of *K*_α_ as a function of $\varepsilon_{A}$ for the porcine organs, and it can be observed that there appears to be a linear relationship with the axial strain. The data was fit to the expression:

$$K_{\alpha }=k_{0}\left(1+b\varepsilon_{A}\right)$$ (6)

where $k_{0}$ is $K_{\alpha }$ as $\varepsilon_{A}=0.$ Table 4 gives the parameters from the linear fit of Eq. 6 in all porcine organs. It was observed that α and η were dependent on the axial strain and Table 5 shows their change with the axial strain.

**TABLE 4:** Parameters from linear fit in the plot of *K*_α_ as a function of $\varepsilon_{A}$ .

Parameters	Heart	Kidney	Liver	Brain
$k_{0}$ (kPa.(s)^α^)	1.94 ± 0.60	0.69 ± 0.20	0.26 ± 0.05	0.13 ± 0.02
b	0.10 ± 0.03	0.23 ± 0.07	0.12 ± 0.02	0.07 ± 0.01

**TABLE 5:** Parameters α and η as a function of $\varepsilon_{A}$ for heart, kidney, liver and brain.

Axial strain (%	Heart		Kidney		Liver		Brain	
	α	η (Pa.s)	α	η (Pa.s)	α	η (Pa.s)	α	η (Pa.s)
0.5	0.13	10.3	0.12	7.58	0.16	9.70	0.16	10.5
2.5	0.11	18.4	0.11	10.9	0.14	13.1	0.15	13.2
4.5	0.11	17.6	0.10	13.5	0.14	13.4	0.15	13.0
6.5	0.11	21.2	0.10	14.3	0.13	16.1	0.15	13.2
8.5	0.11	20.6	0.09	20.1	0.13	14.6	0.15	12.4
10.5	0.10	25.6	0.09	22.1	0.13	17.5	0.15	12.3

### 3.3 Stress relaxation tests

For the stress relaxation test the samples were compressed to a contact force of 0.1 N and at time t = 0, they were subjected to a step shear strain of 0.1%. The shear stress was then measured every second for 10 s. For the S-FKV model, the response to a step strain $\in =\in_{0}H\left(t\right)$ (where H(t) is Heaviside function) results in a relaxation modulus:

$$G\left(t\right)=\frac{\sigma \left(t\right)}{\in_{0}}=\eta \;\delta \left(t\right)+k_{\alpha }\frac{\;t^{-\alpha }}{Ґ\left(1-\alpha \right)}$$ (7)

where Ґ is the gamma function.

**FIGURE 8:**
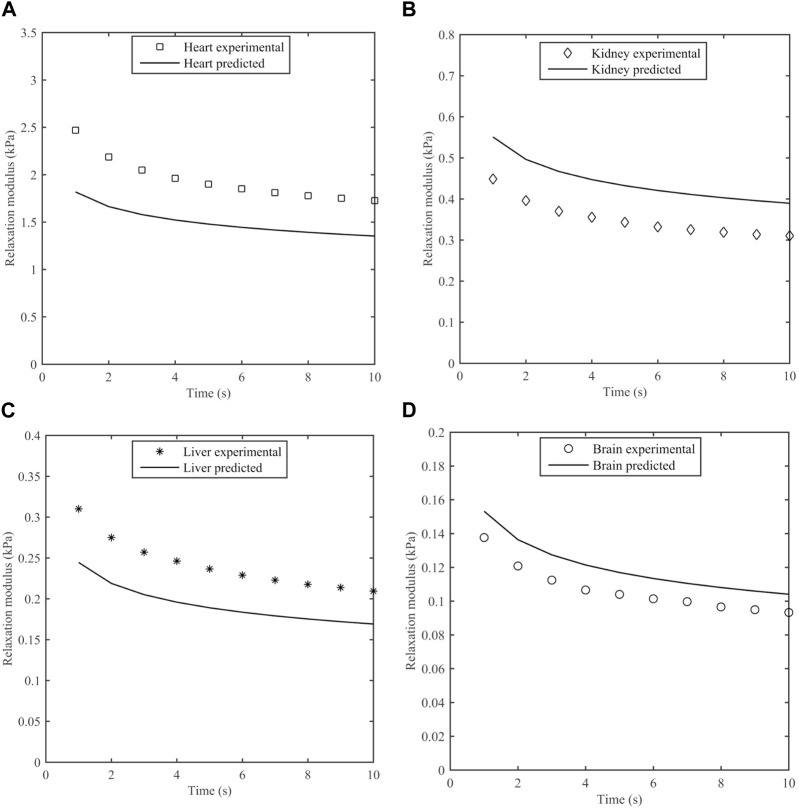
Measured stress relaxation modulus and predicted stress relaxation modulus using S-FKV model for **(A)** Heart, **(B)** Kidney, **(C)** Liver and **(D)** Brain.

The measured stress was compared to predictions based on the S-FKV model using parameters in Table 3. Figure 8 represents the experimental and predicted stress relaxation for different porcine organs. It can be seen that the relaxation time scales of the model and measurements matched well but that the initial values at 1 s varied between the model and measurement. On average, the predicted values were 29% less for heart, 22% greater for kidney, 25% less for liver and 11% greater for brain.

## 4 Discussion

Measurements of the mechanical properties of porcine heart, kidney, liver and brain are reported here. Storage modulus and loss modulus remained steady during strain sweep tests from 0.01% to 0.1% shear strain suggesting that all organs exhibit linear viscoelastic behaviour in this region. Frequency sweep tests were performed at 0.1% shear strain, and a S-FKV model fitted to the frequency sweep data at different axial strains for all the porcine organs with a correlation coefficient better than 0.99. The S-FKV model had a better fit than both the SLS, which couldn’t capture the storage modulus and loss modulus, and the FKV which couldn’t capture the frequency dependence of the loss modulus. We note that including more viscoelastic elements in the SLS, i.e., generating a Prony series (Mishra and Cleveland, 2024), could result in a better fit but at the cost of needing to fit more parameters. Here all the models we compared only had three fitting parameters.

The coefficient of consistence (*K*_α_) increased monotonically with the axial strain for all the organs and there are two potential reasons behind this increase. Firstly, there is a close contact between connective tissue layers during the pre-compression forcing the interstitial fluid out of the tissue matrix and leading to a more solid like behaviour. Secondly, a higher stress is accumulated at small strains in soft tissues due to their hyperelastic nature thereby requiring a higher torque to deform these samples and hence, a higher storage and loss modulus. The stiffness in shear modulus due to compression observed in our study is similar to the result obtained in the literature (Hrapko et al., 2008; Engstrom et al., 2019). The shear modulus in the study (Hrapko et al., 2008) performed on ex-vivo porcine brains increased by 20% as the precompression force was increased from 5 to 10 mN. A compressional study (Engstrom et al., 2019) performed on mammalian brain tissue observed a linear increase in the shear storage modulus with the uniaxial prestress.

The predictive power of the S-FKV model was tested by comparing the predicted response to a step shear strain with the measured response. The stress relaxation amplitudes of the predictions and measurements were within 30%. One potential reason for the discrepancy is that the S-FKV model was fit to data between 0.1 Hz and ∼10 Hz and so may not capture the response outside this range. We note that the decay of the predicted and measured curves in Figure 8 are very similar and the cause of the discrepancy appears to be associated with the initial values, which will be dependent on the high frequency response of the tissue. A second potential reason is that measurements in the frequency and time domains were done on different tissues samples. The standard deviation in the fitted values for K_α_ values were 32% in heart, 28.4% in kidney, 22% in liver and 12% in brain which are similar to the differences between the S-FKV model and the relaxation modulus and so biological variability could also contribute to the differences.

The data reported here were all taken at 20℃ , however living tissue would be at body temperature, normally 37℃ . The effect of this change in temperature was assessed by performing frequency sweep tests on kidney samples immersed in PBS solution at 37℃ by adjusting the peltier plate temperature in rheometer. The shear modulus of kidney samples at 1.2 Hz varied less than 10% as the temperature was increased from 20℃ to 37℃ (see Supplementary Material) and assuming other organs are similar this difference is within 10%.

The fitted S-FKV model was used to predict the shear modulus of organs at frequencies reported in the literature and comparison with literature values are shown in Table 6. It can be seen that in many cases the S-FKV model prediction of shear modulus is consistent with literature values but in some cases there are large discrepancies: up to a factor of 10 is observed in animal organs and up to 27 in human organs. One potential reason is that S-FKV data was measured at frequencies <10 Hz and literature values included measurements up to 1,000 Hz; and the largest discrepancies were typically for data extrapolated beyond 10 Hz. Also, the power law exponent reported here, ∼0.15, is less than reported in the literature 0.3–0.9 (Hoffman et al., 2006; Sack et al., 2009; Grosz et al., 2019; Bonfanti et al., 2020) but again the literature values were obtained in the frequency range of 0.1 Hz–2000 Hz well above the bandwidth measured here. This gives further support to the high-frequency response being responsible for the discrepancies observed in comparison with the relaxation modulus. Other reasons could include variability in the tissue samples and variations in the methods employed.

**TABLE 6:** Shear modulus reported in the literature compared to the S-FKV model prediction with parameters given by Table 3.

Technique	Organ	Study	Frequency (Hz)	Average shear modulus (kPa)	S-FKV storage modulus (kPa)
Magnetic resonance elastography	Porcine heart	Kolipaka et al. (2010)	80	7.69	3.46
Magnetic resonance elastography		Nenadic et al. (2009)	40–500	12.70	3.16–4.39
Rheomtery	Porcine kidney	Nicolle et al. (2010)	0.1–4	2.00	0.66–1.02
Rheomtery		Nasseri et al. (2002)	0.01–20	5.10	0.50–1.24
Magnetic resonance elastography		Kruse et al. (2000)	75–300	1.67	1.45–1.71
Shear wave dispersion ultrasound vibrometry		Amador et al. (2009)	50–500	2.30	1.38–1.82
Shear wave elastography	Porcine liver	Chintada et al. (2020)	100–200	1.39	0.55–0.61
Magnetic resonance elastography		Kruse et al. (2000)	75–300	2.73	0.52–0.65
Rotational rheometer		Nicolle et al. (2010)	0.1	0.80	0.18
Rheological tests		Wex et al. (2013)	0.1–10	0.78	0.18–0.38
Dynamic mechanical testing	Human liver	DeWall et al. (2012)	1–30	3.00	0.26–0.45
Rheometry (time-temperature superposition)	Porcine brain	Shen et al. (2006)	589	1.50	0.67
Oscillatory shear testing		Thibault et al. (1998)	20–200	1.01	0.39–0.57
Oscillatory shear testing (time temperature superposition)		Wismans et al. (1999)	260–1,000	1.63	0.59–0.73
Rotational rheometry		Hrapko et al. (2008)	1–10	0.55	0.24–0.35
Rotational shear testing		Hrapko et al. (2006)	0.04–16	0.52	0.14–0.38
Shear testing (custom-made)		Arbogast et al. (1997)	20–200	1.50	0.39–0.57
Magnetic resonance elastography	Human brain	Sack et al. (2009)	25–62.5	1.56	0.41–0.47
Dynamic shear testing		Fallenstein et al. (1969)	9–10	0.85	0.34–0.35
Dynamic torsion testing		Shuck and Advani (1972)	2–10	7.17	0.27–0.35
Magnetic resonance elastography		Hiscox et al. (2020)	50	2.62	0.45

## 5 Conclusion

In this paper, the rheological behaviour of four porcine organs has been studied from 0.1 Hz up to 9.5 Hz for a range of strain amplitudes and compression. Strain sweep results show that 0.1% strain is within the linear viscoelastic region for all organs. Frequency sweep results showed a monotonic increase in both the storage modulus and loss modulus as a function of frequency and axial strain. This behaviour was better captured with a S-FKV model than a SLS or FKV model. The stress relaxation behaviour of different porcine organs was compared with the stress predicted by S-FKV model and the amplitudes were within 30%. The S-FKV model was extrapolated to predict the shear storage modulus at higher frequencies, and the predictions were consistent with values reported in the literature using other non-invasive methods.

## Data Availability

The original contributions presented in the study are included in the article/Supplementary Material, further inquiries can be directed to the corresponding author.
